# Bezlotoxumab prevents extraintestinal organ damage induced by *Clostridioides difficile* infection

**DOI:** 10.1080/19490976.2022.2117504

**Published:** 2022-08-31

**Authors:** Steven J. Mileto, Melanie L. Hutton, Sarah L. Walton, Antariksh Das, Lisa J. Ioannidis, Don Ketagoda, Kylie M. Quinn, Kate M. Denton, Diana S. Hansen, Dena Lyras

**Affiliations:** a Infection and Immunity Program, Monash Biomedicine Discovery Institute and Department of Microbiology, Monash University, Clayton, Australia; b Cardiovascular Disease Program, Monash Biomedicine Discovery Institute and Department of Physiology, Monash University, Clayton, Australia; c Walter and Eliza Hall Insitiute, Infectious Diseases and Immune Defence Division, Parkville, Australia; d Department of Medical Biology, the University of Melbourne, Parkville, Australia; e RMIT University School of Biomedical and Health Sciences, Chronic Inflammatory and Infectious Diseases Program, Bundoora, Australia

**Keywords:** Clostridioides difficile, toxin, epithelial damage, bacterial infection, systemic disease, colon damage, leaky gut

## Abstract

*Clostridioides difficile* is the most common cause of infectious antibiotic-associated diarrhea, with disease mediated by two major toxins TcdA and TcdB. In severe cases, systemic disease complications may arise, resulting in fatal disease. Systemic disease in animal models has been described, with thymic damage an observable consequence of severe disease in mice. Using a mouse model of *C. difficile* infection, we examined this disease phenotype, focussing on the thymus and serum markers of systemic disease. The efficacy of bezlotoxumab, a monoclonal TcdB therapeutic, to prevent toxin mediated systemic disease complications was also examined. *C. difficile* infection causes toxin-dependent thymic damage and CD4^+^CD8^+^ thymocyte depletion in mice. These systemic complications coincide with changes in biochemical markers of liver and kidney function, including increased serum urea and creatinine, and hypoglycemia. Administration of bezlotoxumab during *C. difficile* infection prevents systemic disease and thymic atrophy, without blocking gut damage, suggesting the leakage of gut contents into circulation may influence systemic disease. As the thymus has such a crucial role in T cell production and immune system development, these findings may have important implications in relapse of *C. difficile* disease and impaired immunity during *C. difficile* infection. The prevention of thymic atrophy and reduced systemic response following bezlotoxumab treatment, without altering colonic damage, highlights the importance of systemic disease in *C. difficile* infection, and provides new insights into the mechanism of action for this therapeutic.

**Abbreviations**: Acute kidney injury (AKI); Alanine Transaminase (ALT); Aspartate Aminotransferase (AST); *C. difficile* infection (CDI); chronic kidney disease (CKD); combined repetitive oligo-peptides (CROPS); cardiovascular disease (CVD); Double positive (DP); hematoxylin and eosin (H&E); immunohistochemical (IHC); multiple organ dysfunction syndrome (MODS); phosphate buffered saline (PBS); standard error of the mean (SEM); surface layer proteins (SLP); Single positive (SP); wild-type (WT).

## Introduction

*Clostridioides difficile* is the most commonly identified infectious cause of antibiotic-associated diarrhea in developed countries, with infection resulting in a variety of disease symptoms ranging from asymptomatic carriage, mild and self-limiting diarrhea, to severe diarrhea with potential life-threatening complications.^1^ Disease is toxin-mediated and results from the production and activity of two major toxins, TcdA and TcdB, following vegetative cell colonization of the anaerobic large intestine.^1^ A third toxin, CDT, present in roughly one third of clinical isolates,^2,3^ is also implicated in disease, with a suspected role in enhancing the damage induced by TcdA and TcdB and aiding in colonization.^4,5^ Disease presentation during *C. difficile* infection (CDI) may be influenced by the toxin profile or clade of a given strain.^6^ Notably, TcdB appears to be more prevalent amongst strains and has many variant forms, some of which are encoded by epidemic human and animal strains, and may induce more severe cytopathic effects *in vitro*.^7–12^ TcdB from ribotype 027 strains, which have contributed to several outbreaks of severe disease over the last two decades, also appears dissimilar to historical TcdB, with variations in the combined repetitive oligo-peptides (CROPS) domain.^13^ Notably, treatment of embryonic zebrafish with ribotype 027 TcdB induced more severe and extensive pathologies than historical TcdB, suggesting variations in TcdB may alter tissue tropism.^13^

The differences in TcdB activity and propensity to induce more severe disease in recent years have highlighted a need to understand the exact role of toxins in CDI and disease progression. In severe cases of CDI, including pseudomembranous colitis or toxic megacolon, sepsis and patient mortality may occur as a result of severe and detrimental inflammation and swelling of the colon, with colonic wall thickening.^1^ These severe pathologies can be compounded by systemic disease complications, which presumably result from erosion of the epithelial layer of the colon, allowing for the leakage of gut contents, including microbiota, *C. difficile* and its toxins, into the blood and lymphatic systems.^14–16^ Following the damage and loss of epithelial integrity, both TcdA and TcdB may escape the colonic barrier and enter the bloodstream, ascites or pleural fluid, resulting in toxemia which may lead to disease pathology in organs external to the colon.^17,18^

Bacterial translocation and lipopolysaccharide escape during gut infections, resulting from increased intestinal permeability, has also been implicated in the development of sepsis and septic shock leading to multiple organ dysfunction syndrome (MODS),^19,20^ and is associated with an increased risk of cardiovascular disease (CVD), acute kidney injury (AKI) and chronic kidney disease (CKD).^21,22^ Since patient co-morbidities such as CVD and CKD can increase CDI risk and mortality,^23,24^ these diseases may also perpetuate one another. The advanced age of most CDI patients (>65 years;^25^) adds to this risk for severe disease, with immunosenescence (deterioration of immune function with age) a likely contributing factor. During severe CDI, damage to the colon and disease complications may be compounded by the onset of MODS, further increasing patient fatality.^26^ We recently showed that the thymus undergoes changes during severe CDI in mice, with toxin-mediated disorganization of the cortex and medulla and thymic apoptosis observed.^27^ The increasing incidence of CDI, and the heightened disease severity associated with ribotype 027, 017, 126 and 244 *C. difficile* strains, among others, has resulted in more cases with severe disease and life-threatening complications.^7,10–12,28,29^ However, our understanding of these systemic complications during CDI is limited, and this aspect of disease is often overlooked.

We aimed to further characterize thymic damage during CDI, and to identify the precise changes that occur during infection-mediated thymic atrophy. Furthermore, we hypothesized that systemic disease during toxigenic CDI would lead to detectable changes in kidneys and serum markers, and that treatment with toxin-specific therapeutics, such as bezlotoxumab, would prevent the development of these systemic pathologies. Here, we show that CDI in mice induces toxin-dependent thymic and kidney inflammation and damage, depleting the thymus of CD4^+^CD8^+^ thymocytes, which are critical for replenishing the circulating T cell pool. This thymic and kidney damage coincides with changes to systemic damage serum markers including urea, creatinine, and glucose, as well as physiological and behavioral changes consistent with systemic disease. Administration of bezlotoxumab during CDI blocked systemic disease complications, thymic atrophy and kidney inflammation, but did not prevent gut damage. These results suggest that toxin-mediated damage to the epithelial barrier may contribute to disease outside of the colon through the dissemination of luminal contents beyond the gut, and that targeting this aspect of infection unexpectedly reduces overall disease severity. This work provides a new mechanistic understanding of how bezlotoxumab may improve patient outcomes during CDI, and uncovers unappreciated contributions of systemic infection to disease outcomes.

## Materials and methods

### Bacterial culture conditions and strains

The *C. difficile* parental strain M7404 (Ribotype 027; TcdA^+^TcdB^+^CDT^+^) and the isogenic nontoxigenic mutant strain DLL3121 (TcdA^−^TcdB^−^CDT^+^) were cultured and spores subsequently prepared for animal infections as described previously.^30^ While DLL3121 does not produce TcdA and TcdB, it still produces CDT, however it is incapable of inducing severe disease, as previously shown.^27^

### Animal infections

Animal handling and experimentation was performed according to Victorian State Government regulations, approved by the Monash University Animal Ethics Committee (Monash University AEC no. MARP/2014/135 and 17096). Male, six to seven-week-old, C57BL/6 J mice (Walter and Eliza Hall Institute of Medical Research) were pre-treated with an antibiotic cocktail in the drinking water for seven days, followed by three days of cefaclor alone, as previously described.^30^ Antibiotic treatment ceased on the day of infection. For the time-course comparison of the *C. difficile* strains M7404 and DLL3121, mice were infected with 10^6^ spores of a single strain of *C. difficile* by oral gavage, or left uninfected as controls, and monitored twice daily for signs of distress, in addition to weight loss and fecal consistency, as described previously.^30^ For the evaluation of bezlotoxumab (Zinplava ™) as a preventative for systemic disease, mice were infected with 10^6^ spores of *C. difficile* M7404 by oral gavage. Twenty-four hours prior to infection or 24 hours post infection, mice were administered a single dose of either bezlotoxumab (10 mg/kg), or phosphate buffered saline (PBS) (100 µL) as a control *via* intraperitoneal injection. Efficacy of PBS as a control was also compared to use of a human IgG1 isotype control (BioX Cell; 10 mg/kg), administered as a single dose 24 hours prior to infection, *via* intraperitoneal injection in initial experiments. Mice were euthanized upon reaching defined ethical endpoints or at the end of the experiment, two days following infection, as described previously.^30^ To ascertain the efficacy of bezlotoxumab in preventing systemic disease complications, mice were scored for changes in physiological activity and appearance, using a murine sepsis severity scoring protocol modified from Shrum *et al*^31^ with modifications as follows; Appearance: coat is smooth (0), patches of hair piloerected (1), majority of back is piloerected (2), piloerection may or may not be present, mouse appears “puffy” (3), or, piloerection may or may not be present, mouse appears emaciated (4); Activity: normal activity/mouse is: eating, drinking, climbing, running, fighting (0), slightly suppressed activity/mouse is moving around bottom of cage (1), suppressed activity/mouse is stationary with occasional investigative movements (2), no activity/mouse is stationary (3), or, no activity/mouse experiencing tremors, particularly in the hind legs (4); Eyes: eyes open (0), eyes not fully open, possibly with secretions (1), eyes half closed, possibly with secretions (2), eyes half closed or more, possibly with secretions (3), or, eyes closed or milky (4); Respiration: normal breathing (0), brief labored breathing (1), labored breathing without gasping (2), labored breathing with intermittent gasps (3), or, gasping (4). For all experiments, fecal samples were collected from each mouse at 24 hours post-infection and when euthanized, resuspended in PBS (100 mg/mL), heat shocked (30 minutes, 65°C) and plated as described previously^30^ to assess colonization of the infecting strain.

### Histopathological scoring and staining

Four-micron sections of formalin fixed (10%, Neutral buffered), Swiss-rolled colon were periodic acid–Schiff/Alcian blue stained and assessed using a previously described scoring system.^27^ Thymic tissue was fixed in either formalin or snap frozen in Optimal Cutting Temperature (O.C.T) compound (Tissue-Tek®) and 4 μm sections stained with hematoxylin and eosin (H&E). Kidneys were fixed in 4% paraformaldehyde and sectioned for immunohistochemical (IHC). Paraffin embedded kidneys were cut into 4 μm sections before dewaxing using standard procedures, and antigen retrieval was performed using 10 mM citrate (Sigma-Aldrich) buffer, with 0.05% Tween-20 (Amresco), pH 6.0. Endogenous peroxidases were then quenched with 0.3% hydrogen peroxide (Sigma-Aldrich), for 30 minutes. Slides were blocked for 30 minutes with goat serum (1:10 diluted with antibody diluent (DAKO)) at room temperature. Slides were then incubated with rabbit anti-F4/80 (Cell Signaling Technologies; 1:500 dilution in antibody diluent (DAKO)) for 60 minutes at room temperature, before rinsing three times in PBS-T (0.1% Tween 20) and incubating with the biotinylated goat anti-rabbit secondary antibody (Vector Labs; 1:200 dilution in antibody diluent (DAKO)) for 45 minutes at room temperature. Slides were rinsed 3 times, before incubating with VECTASTAIN Elite ABC Reagent for 30 minutes, at room temperature. Slides were again rinsed 3 times, before adding the DAB peroxidase substrate solution (Vector Labs), rinsing once color had developed. Slides were counterstained with hematoxylin and dehydrated and cover slipped using standard procedures. H&E and IHC stained sections were scanned for visualization using an Aperio Scanscope AT Turbo, at 20 times magnification. For each kidney, 30 glomeruli were image and counted for F4/80+ cells. All histopathological analysis was performed on de-identified specimens.

### Colonic cytokine arrays

Colonic tissue was homogenized in 300 μL of lysis buffer (1% Triton-X 100 (Amresco), in PBS, with one protease inhibitor cocktail tablet/20 mL (cOmplete™, EDTA-free Roche)) and centrifugation at 10,000 *g* for five minutes at 4°C. The protein content of the supernatant of each lysate was determined using a BCA assay (Thermo Scientific: Pierce®) and was adjusted to 200 µg of protein and then used for cytokine analysis using the Proteome ProfilerTM Array, Mouse Cytokine Array Panel A (R & D systems), following the manufacturer’s instructions. Cytokine array membranes were exposed to an X-ray film, processed and scanned. Signal intensity was analyzed by densitometry using Image J software, following the removal of background noise and color inversion. A fixed size circle was used to measure signal intensity, subtracting the negative control intensity from each sample. Signal intensities were averaged and plotted using GraphPad Prism 9.

### Serum cytokine arrays, IL-6 ELISAs and biochemical marker analysis

Blood was collected from mice post mortem *via* cardiac bleed, and left to clot. The resulting supernatant was centrifuge at 1,000 *g* for 10 minutes at 4°C, using the cleared serum for subsequent analysis.

Serum collected from the time course of infection was analyzed using a Quantibody Mouse Custom multiplex Array (Crux Biolabs) analyzing GCSF, IL-1β, IL-3, IL-6, CXCL1, CXCL9, MCP-1, TNF-α and TREM-1. Serum was diluted two-fold before preparation of the samples for analysis as per the manufacturer’s instructions. Samples were run and analyzed on a Bio-Plex® MAGPIX™ Multiplex Reader (BioRad).

Serum collected for the biochemical markers aspartate aminotransferase (AST; ab105135), alanine transaminase (ALT; ab105134), creatinine (ab65340), urea (ab3362), and glucose (ab65333) were measured using commercially available diagnostic kits (Abcam) as per the manufacture’s protocol, in duplicate. Plates were analyzed on an automated plate reader (Tecan) at the appropriate wavelength as per the assay protocol. Samples used for the glucose assay kit required deproteinization prior to the conduction of the assay. These samples were trichloroacetic acid precipitated (4 M) and neutralized with potassium hydroxide (2 M), centrifuging at 10,000 *g* for 10 minutes at 4°C to remove protein contaminants. The percentage of serum in the precipitated samples was calculated and used to infer the final glucose concentration. Sample analyte concentrations were calculated from each standard curve.

Serum collected for bezlotoxumab experiments were also analyzed using a mouse IL-6 ELISA (Abcam). Serum was diluted either four-fold (uninfected) or eight-fold (infected) and analyzed in duplicate, as per the manufacturer’s instructions.

### Flow cytometric analysis of the thymus

Thymic cells (1x10^6^) were then stained with rat anti-mouse CD4-FITC (BD Biosciences; 1:400) and rat anti-mouse CD8a-PE (BD Biosciences; 1:400) and rat anti-mouse CD45-PerCP-Cy™5.5 (BD Biosciences; 1:400) for 30 minutes on ice. Cells were washed prior to analysis, resuspended in FACS buffer and analyzed using a FACSCalibur flow cytometer or a BD Fortessa X-20 FACS machine, gating on CD45; with data analysis conducted using CellQuest Pro software (BD Biosciences) and FlowJo 10.2, respectively.

### Thymocyte and splenocyte killing assays

Thymocytes and splenocytes isolated from three-month-old male C57BL/6 J mice (MARP) were seeded at 1 × 10^6^ cells per well in RPMI supplemented with 2 mM L-glutamine, 1 mM MEM sodium pyruvate, 100 uM MEM non-essential amino acids, 5 mM HEPES buffer solution, 55 uM 2-mercaptoethanol, 10 U/mL IL-2, 100 U/mL penicillin and 100 ug/mL streptomycin. Cells were exposed to either purified TcdB from strain VPI10463 (Abcam) diluted to 80 ng/mL, 16 ng/mL, 3.2 ng/mL, 0.64 ng/mL and 0.128 ng/mL, or 10% DMSO or 1 μM oligomycin (as high or moderate positive controls to induce cell death in leukocytes), for 20 hours, using untreated cells as a control, before imaging cells on an EVOS FL Auto Cell Imaging System (Invitrogen). Following cell imaging, the cell surfaces were stained with LIVE/DEAD™ Fixable Aqua (1:400; Invitrogen), Propidium Iodide (1:800; BD Biosciences), anti-mouse CD3-PE (1:400; BD Biosciences), anti-mouse CD4-AF700 (1:400; Biolegend), and anti-mouse CD8-PacBlue (1:400; BD Biosciences) antibodies for 15 minutes, in the dark, on ice. Cells were washed three times, before resuspending in 200 µL of FACS buffer, and quantified using a BD LSRII Flow Cytometer (BD Biosciences). Analysis was performed on FlowJo version 9.9.6.

### Vero cell killing assays

Vero cells were cultured and prepared as previously described.^30^ Cells were seeded in 96-well plates at 1 x 10^4^ cells/well, and incubated for 24 hours at 37°C in 5% CO_2_. Cells were exposed to either purified TcdB from strain VPI10463 (Abcam) diluted to 80 ng/mL, 16 ng/mL, 3.2 ng/mL, 0.64 ng/mL and 0.128 ng/mL, or 10% DMSO or 10% oligomycin, for 20 hours, using untreated cells as a control, before imaging cells on an EVOS FL Auto Cell Imaging System (Invitrogen). Following cell imaging, detached cells were removed and quantified using a BD LSRII Flow Cytometer (BD Biosciences). Analysis was performed on FlowJo version 9.9.6.

### Data analysis and statistics

Data were plotted (as averages, with error presented as standard error of the mean (SEM)) using GraphPad Prism 9, with statistical analyses conducted using a Mann Whitney *U* test.

## Results

### Toxigenic CDI induces systemic inflammation and extra-intestinal organ damage

**Figure 1.**
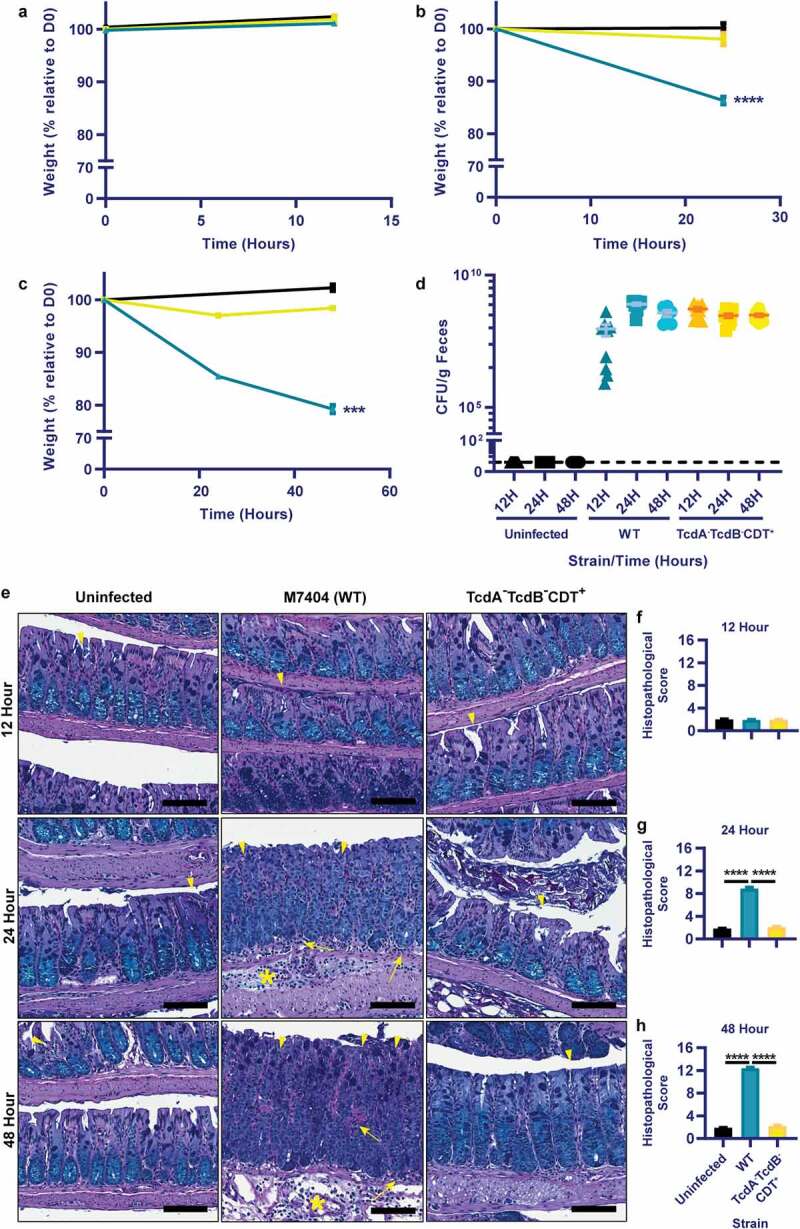
Toxigenic M7404 (WT) *C. difficile* infection leads to weight-loss and colonic damage but the TcdA^−^TcdB^−^CDT^+^ strain does not, despite similar colonization levels. Groups of C57BL/6 J mice (n = 10 per group) were infected with either M7404 (WT) or TcdA^−^TcdB^−^CDT^+^ *C. difficile* and monitored for weight-loss and spore shedding in feces daily. Uninfected control mice were also monitored. Mice were euthanized at 12-, 24- or 48-hours post-infection. Weight-loss relative to day zero (D0) was plotted at 12- (a), 24- (b), and 48- (c) hours post-infection. d) Spore shedding was enumerated at each time point, with spore counts plotted following anaerobic growth for 48-hours at 37°C. Dotted line represents the limit of detection for spore enumeration. Colonic tissue was removed, Swiss-rolled and fixed prior to sectioning and Periodic Acid Schiff/Alcian Blue staining. e) Representative images of colonic histopathology from WT or TcdA^−^TcdB^−^CDT^+^ *C. difficile*-infected mice, or from uninfected mice. Sections of colonic tissue from WT (blue) or TcdA-TcdB-CDT+ (yellow) *C. difficile*-infected mice or from uninfected animals (black) were scored using four different tissue damage parameters, providing a cumulative histopathological score, and plotted for 12- (f), 24- (g), and 48- (h) hours post-infection. Error bars indicate S.E.M. *** = p ≤ .001; **** = p ≤ .0001. Scale bar = 100 µm. Arrow = Inflammation; Arrowhead = Crypt damage, or goblet cell loss; Asterisk = Edema.

The understanding of systemic complications during CDI is limited. To further characterize this disease aspect, a mouse model of CDI was employed. Uninfected mice or mice infected with a wild-type (WT) *C. difficile* (M7404) strain or an isogenic mutant strain that does not produce TcdA and TcdB (DLL3121, TcdA^−^TcdB^−^CDT^+^) were compared at 12, 24- and 48-hours post-infection. As expected, disease severity, measured by weight loss and gut pathology, correlated with the presence of TcdA and TcdB, and worsened with progression of infection over time in the WT strain, with disease absent in both the TcdA^−^TcdB^−^CDT^+^ and uninfected groups (Figure 1). Little to no weight loss was seen in the first 12-hours of infection with either strain (Figure 1a), however, by 24-hours post-infection, mice infected with WT bacteria had lost >10% weight loss relative to day zero compared to ~2% weight loss caused by the TcdA^−^B^−^CDT^+^ strain (*p*< .0001; Figure 1b). At 48-hours post-infection, disease was severe in mice infected with the WT strain, with mice reaching ~20% weight loss relative to day zero (*p*= .0001; Figure 1c). Infection with the TcdA^−^TcdB^−^CDT^+^ mutant resulted in little weight loss at each time point, despite similar levels of *C. difficile* being shed in comparison to the WT strain at each time point (Figure 1d). The weight loss detected in the WT-infected mice was reflected in the high levels of colonic damage that were observed, which were characterized by large regions of crypt damage, hyperplasia, goblet cell loss and inflammation, and edema of the mucosa and sub-mucosa (Figure 1e). Furthermore, a significant increase in colonic pathology damage scores was seen at both 24- and 48-hours post-infection in WT-infected mice when compared to both the TcdA^−^TcdB^−^CDT^+^ group and uninfected control mice (*p*= .0001; Figure 1f-h).

Severe colonic damage in the WT-infected group was associated with increased colonic C5a, GCSF, IL-1β, IL-2, IL-3, CXCL1, CXCL9, CXCL10, MCP-1, TIMP-1, TNF-α and TREM expression upon the development of gut pathologies at 24- and 48-hours, compared to the TcdA^−^TcdB^−^CDT^+^ and uninfected groups (Supplementary Figure 1a-c, *p*< .05). Heightened colonic cytokine levels in the WT-infected group also correlated with significant increases in serum GCSF, IL-1β, IL-3, IL-6, IL-17, CXCL1, CXCL9, MCP-1, TNF-α and TREM at the peak of infection (48-hours), when compared to the TcdA^−^TcdB^−^CDT^+^ and uninfected groups (Supplementary Figure 1d, *p*< .05).

**Figure 2.**
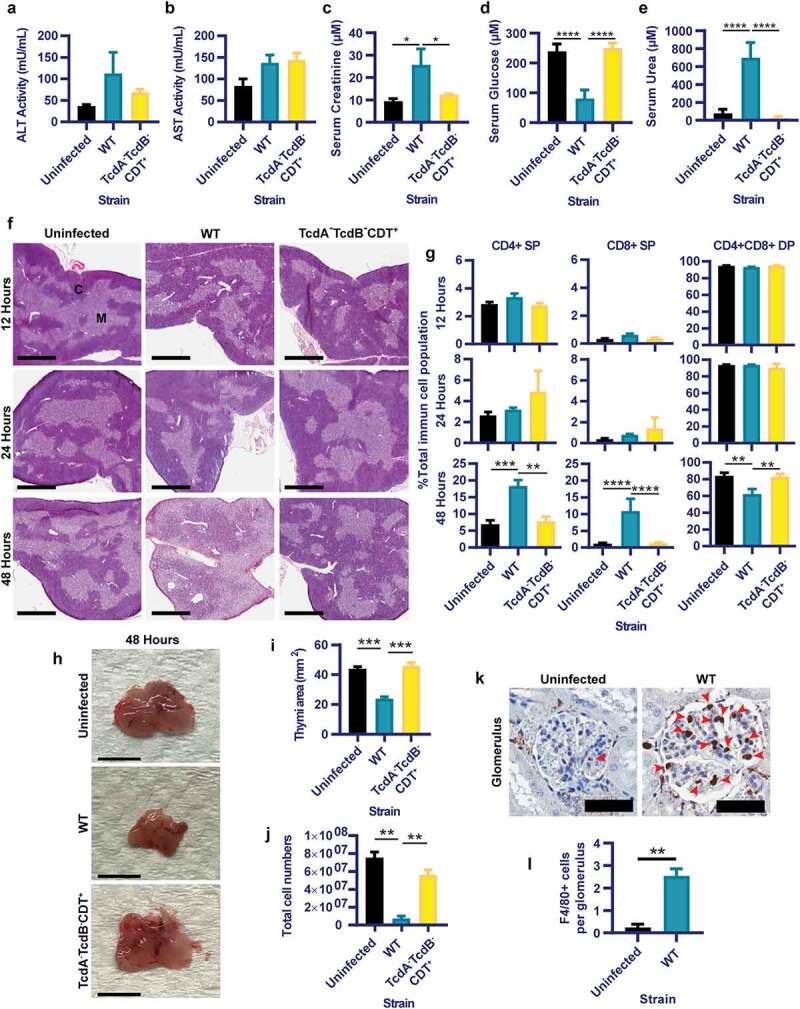
Toxigenic M7404 (WT) *C. difficile* infection results in systemic organ complications, including thymic and kidney damage, in a toxin and time dependent manner. C57BL/6 J mice (n = 5) were infected with WT or TcdA^−^TcdB^−^CDT^+^ *C. difficile* or left uninfected then euthanized at 12-, 24- or 48-hour time-points. Serum was collected at 48-hours post infection and analyzed for (a) alanine transaminase (ALT), (b) aspartate aminotransferase (AST), (c) Creatinine, (d) Glucose and (e) Urea. f) Representative images of hematoxylin and eosin-stained thymic sections from WT or TcdA^−^TcdB^−^CDT^+^ *C. difficile-*infected or uninfected mice at 12-, 24- and 48-hour time-points. C = Cortex; M = Medulla. Scale bar = 1 mm. g) Single positive (SP) CD4^+^ and CD8^+^ T cell populations as well as double positive (DP) CD4^+^CD8^+^ thymocytes in the thymus at 12-, 24- and 48-hour time-points from WT (blue) or TcdA^−^TcdB^−^CDT^+^ (yellow) infected and uninfected (black) mice as determined by FACS analysis. Data is presented as the percentage of each of these T cell populations of the total immune cell population. h) Representative images of whole thymi, (i) total thymic area and (j) total thymic cell numbers. Scale bar = 5 mm. k) Representative images of macrophage (F4/80 + cells) infiltration into kidney glomeruli, with l) average counts plotted. Scale bar = 50 µm; Arrow = Macrophage. Error bars indicate S.E.M. Statistical analysis by Mann Whitney *U* test. * = p ≤ .05; ** = p ≤ .01; *** = p ≤ .001; **** = p ≤ .0001.

To establish if toxin-mediated colonic damage, inflammation and permeability were linked with extra-intestinal complications, sera were assessed from the panel of infected and uninfected mice for markers of systemic damage. Although significant hepatocyte damage following toxigenic colonic damage was not apparent, indicated by similar levels of liver enzyme Alanine Transaminase (ALT) and Aspartate Aminotransferase (AST)^32^ activity in the serum of all mice (Figure 2a, b), significant differences were detected for serum creatinine, glucose and urea for WT-infected mice when compared to the TcdA^−^TcdB^−^CDT^+^ and uninfected groups (Figure 2c-e). Thus, toxin-mediated colonic damage and gut permeability during CDI may alter kidney creatinine and urea handling,^33,34^ indicated by serum creatinine and urea of WT-infected mice being over twice that detected for the TcdA^−^TcdB^−^CDT^+^ and uninfected groups (*p*< .05; Figure 2c, e). Additionally, severe hypoglycemia in WT-infected mice was detected when compared to the TcdA^−^TcdB^−^CDT^+^ and uninfected groups (*p*< .05; Figure 2d), which may indicate poor liver gluconeogenesis,^35^ or bacteremia.^36,37^

To further examine the extent of systemic disease during CDI, thymi were collected from infected and uninfected mice at early, mid and peak of infection time points (12-, 24- and 48-hours, respectively). The thymus was used as a surrogate measure for systemic pathologies, since the thymus is highly sensitive to systemic inflammatory responses in several infections including *Salmonella* species,^38^ *Trypanosoma cruzi*^39^ and *Plasmodium chabaudi*;^40^ and has been previously shown to undergo damage during CDI.^27^ Additionally, kidneys were collected at the peak of infection (48-hours) from WT-infected and uninfected mice, to assess if increased serum urea and creatinine were linked to pathophysiological changes within this organ.

Histopathological analysis revealed that thymic atrophy, specifically loss of cortex volume and cortex/medulla disorganization, were linked to fulminant disease and toxin-mediated colonic damage at 48-hours post WT-infection (Figure 2f). This correlated with a significant reduction in the frequency of double positive (DP) CD4^+^CD8^+^ thymocytes (37.4% reduction compared to TcdA^−^TcdB^−^CDT^+^-infection and a 38.1% reduction when compared to uninfected mice; *p*= .0079; Figure 2g) with a reciprocal increase in the frequency of single positive (SP) CD4^+^ and CD8^+^ T-cell populations following WT-infection compared to the TcdA^−^TcdB^−^CDT^+^-infected mice (14.5% and 16.44% proportional shift, respectively) and uninfected mice (15.04% and 16.51% proportional shift, respectively). These proportional shifts in DP and SP T-cells were likely compounded by reduced thymic size (*p*= .0003; Figure 2h, i) and total cell numbers (*p*= .0079; Figure 2j), post WT-infection; which has been seen during other infections.^40–44^ Furthermore, when examining kidney glomeruli for macrophage infiltration (as indicated by F4/80+ cells), WT-infection resulted in a significant influx of macrophages into the glomeruli when compared to uninfected mice (*p = *.0079; Figure 2k, l).

### Prophylactic administration of bezlotoxumab prior to infection inhibits CDI mediated extra-intestinal organ damage

Given that the systemic pathologies of CDI, namely elevated cytokine release and organ damage, were linked to toxin-mediated colonic damage, the use of toxin specific therapies was investigated to determine their suitability for the prevention of systemic effects during CDI. Bezlotoxumab (Zinplava©; a monoclonal TcdB therapeutic) provides a non-antibiotic therapeutic approach to CDI treatment, with efficacy in preventing disease relapse.^45–47^ However, the therapeutic value of bezlotoxumab in preventing systemic and severe disease complications during CDI has only been explored with respect to lung damage.^48^ Thus, the therapeutic value of bezlotoxumab in preventing thymic and kidney damage was examined. Changes in markers of systemic damage were also assessed when bezlotoxumab was used prophylactically or as a treatment, in the context of primary infection. Clinical trials testing the efficacy of bezlotoxumab in preventing *C. difficile* recurrence have to date used saline (vehicle alone) as a placebo.^45–47^ Thus, for consistency with clinical data, changes in disease profile following bezlotoxumab treatment were compared to vehicle alone (PBS), after first confirming that isotype control antibodies did not alter systemic disease during CDI and were comparable to PBS treatment of infected mice. Human isotype control IgG1 was administered to mice, *via* I.P injection, 24-hours prior to CDI (or PBS as a vehicle control). Mice were then infected with WT *C. difficile*, and monitored for weight-loss, colonic pathologies and damage to the thymus. Unsurprisingly, no difference was observed between mice treated with either the isotype control or PBS with respect to weight, with both groups of mice observing a weight-loss of >15% relative to day zero within 48-hours of infection (Supplementary Figure 2a). This weight-loss correlated with similar levels of colonic and thymic damage between the two groups, characterized by large regions of colonic epithelial damage, inflammation and edema and a disorganization of the thymic cortex and medullary regions (Supplementary Figure 2b). Colonic histopathological scoring was also similar in each infected group (Supplementary Figure 2c), suggesting that administration of human IgG1 to mice prior to CDI does not alter *C. difficile* disease progression, and is comparable to PBS as a vehicle control.

**Figure 3.**
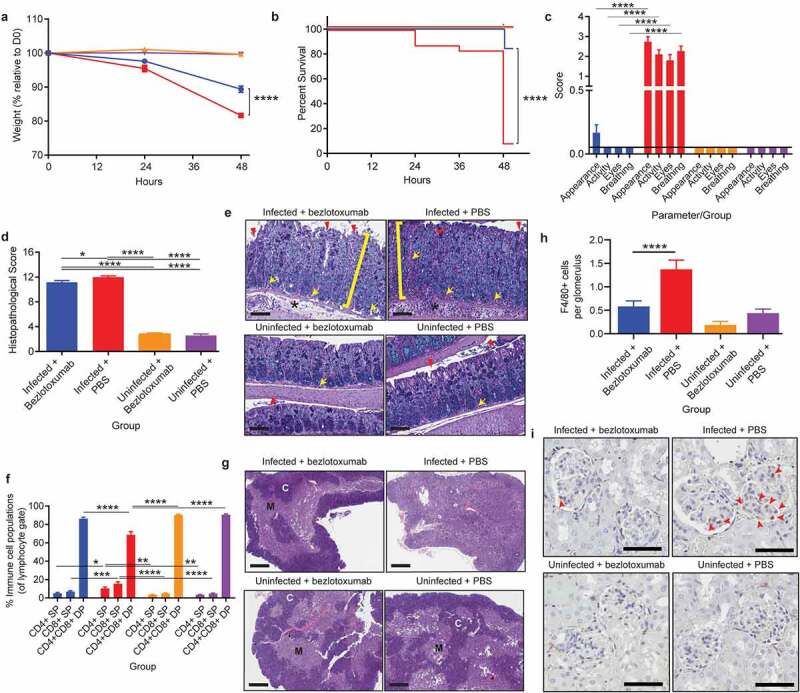
Prophylactic administration of TcdB neutralizing antibody bezlotoxumab increases survival and prevents systemic disease in CDI, without blocking colonic damage. C57BL/6 J mice (n = 10) were intraperitoneally administered either bezlotoxumab (10 mg/kg) or PBS 24-hours prior to infection with M7404 *C. difficile* and were compared to uninfected control mice. a) Weight-loss relative to day zero (D0) and b) Kaplan Meier survival curve of infected + bezlotoxumab (blue), infected + PBS (red), uninfected + bezlotoxumab (Orange) and uninfected + PBS mice (purple). c) Representative scoring of murine sepsis parameters for changes in appearance, activity, eyes and breathing. d) Cumulative colonic histopathology score of four different tissue damage parameters. e) Representative images of Periodic Acid Schiff/Alcian Blue stained Swiss-rolled colonic tissues. Scale bar = 100 µm. Bracket = Crypt hyperplasia, damage or hemorrhage; Yellow arrow = Inflammation; Red arrowhead = Crypt damage or goblet cell loss; Black star = Edema. f) FACS analysis of single positive (SP) CD4^+^ and CD8^+^ T cell populations as well as double positive (DP) CD4^+^CD8^+^ thymocytes in the thymus following CDI and bezlotoxumab intervention. g) Representative images of hematoxylin and eosin-stained thymic sections. C = Cortex; M = Medulla. Scale bar = 1 mm. h) Average counts of macrophage (F4/80 + cells) infiltration into kidney glomeruli, with i) representative images shown. Scale bar = 50 µm; Red arrow = Macrophage. Error bars indicate S.E.M. Statistical analysis by Mann Whitney *U* test. * = p ≤ .05; ** = p ≤ .01; *** = p ≤ .001; **** = p ≤ .0001.

Prophylactic administration of bezlotoxumab, 24-hours prior to CDI, significantly reduced infection mediated weight loss by 8% (~81% of starting weight in infected untreated mice compared to ~89% in treated infected mice; *p*< .0001; Figure 3a), resulting in a 76% increase in survival, when comparing infected mice administered bezlotoxumab to mice that received PBS alone (*p*< .0001; Figure 3b). Bezlotoxumab administration also ameliorated physiological presentations of CDI. Infected mice administered PBS appeared scruffy, had low activity, squinted eyes and an altered breathing pattern, which was significantly different to mice infected following bezlotoxumab exposure, which exhibited a similar coat appearance, activity, eye appearance and breathing pattern to that of uninfected mice (*p*< .0001; Figure 3c). Strikingly, bezlotoxumab did not prevent severe colonic damage during CDI, characterized by significant epithelial damage, inflammation and edema, when compared to uninfected mice (*p*< .0001; Figure 3d, e), and only a mild reduction in colonic inflammation when compared to infected mice administered PBS (*p*= .023; Figure 3d, e).

Although the prophylactic administration of bezlotoxumab did not reduce gut damage, it prevented the development of systemic disease pathologies during CDI. Mice infected with WT *C. difficile* following exposure to bezlotoxumab did not develop thymic atrophy, characterized by a clearly defined cortex and medulla, similar to that seen in uninfected mice (Figure 3f, g). The lack of thymic atrophy in bezlotoxumab treated infected mice also correlated with normal levels of DP thymocytes, SP CD4^+^ T cells and SP CD8^+^ T cells, similar to the control groups (Figure 3f, g). Without treatment, as in the case of infected mice administered PBS, the thymus atrophies, with an indistinguishable cortex and medulla (Figure 3f) and a significant DP thymocyte depletion when compared to the remaining groups (p < .0001; Figure 3g). Both the SP T cell populations increased in the infected mice administered PBS, which again is likely to be linked to a proportional change in cell numbers due to the significant reduction in thymic output and sensitive nature of the CD4^+^CD8^+^ thymocyte population.

Kidney inflammation was also prevented in infected mice administered bezlotoxumab, with glomerular macrophage counts similar to that seen in uninfected mice, compared to infected mice that received PBS in which high macrophage infiltration into the glomeruli (*p*< .0001; Figure 3h, i) was detected. Collectively, these data suggest that the reduced severity of disease in the bezlotoxumab-treated mice may result from a block in disease pathways that occur beyond the colonic epithelial lining. Serum samples were also analyzed for changes in glucose, urea, creatinine and IL-6 levels, which are commonly used markers of murine sepsis; these were all altered in WT-infected mice (Figure 2, Supplementary Figure 2). Prophylactic administration of bezlotoxumab prevented infection-mediated hypoglycemia, uremia, and a spike in IL-6 when compared to infected mice that received PBS (*p = *.0159; Supplementary Figure 2e-g), and a moderate, yet not significant reduction in creatinine levels compared to infected mice that received PBS (Supplementary Figure 2d). These results suggest that bezlotoxumab is able to prevent the induction of systemic complications during CDI when administered prophylactically.

### Administration of bezlotoxumab as a post-infection treatment inhibits CDI mediated extra-intestinal organ damage

**Figure 4.**
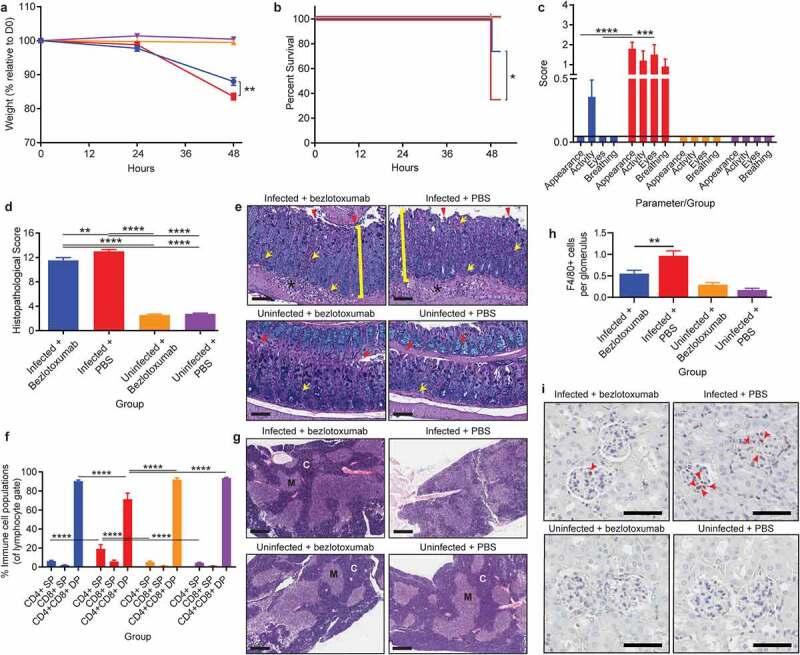
Treatment with TcdB neutralizing antibody bezlotoxumab 24 hours post infection increases survival and prevents systemic disease in CDI, without blocking colonic damage. C57BL/6 J mice (n = 10) were intraperitoneally administered either bezlotoxumab (10 mg/kg) or PBS 24-hours post infection with M7404 *C. difficile* and were compared to uninfected control mice. a) Weight-loss relative to day zero (D0) and b) Kaplan Meier survival curve of infected + bezlotoxumab (blue), infected + PBS (red), uninfected + bezlotoxumab (Orange) and uninfected + PBS mice (purple). c) Representative scoring of murine sepsis parameters for changes in appearance, activity, eyes and breathing. d) Cumulative colonic histopathology score of four different tissue damage parameters. e) Representative images of Periodic Acid Schiff/Alcian Blue stained Swiss-rolled colonic tissues. Scale bar = 100 µm. Bracket = Crypt hyperplasia, damage or hemorrhage; Yellow arrow = Inflammation; Red arrowhead = Crypt damage or goblet cell loss; Black star = Edema. f) FACS analysis of single positive (SP) CD4^+^ and CD8^+^ T cell populations as well as double positive (DP) CD4^+^CD8^+^ thymocytes in the thymus following CDI and bezlotoxumab intervention. g) Representative images of hematoxylin and eosin-stained thymic sections. C = Cortex; M = Medulla. Scale bar = 1 mm. h) Average counts of macrophage (F4/80 + cells) infiltration into kidney glomeruli, with i) representative images shown. Scale bar = 50 µm; Red arrow = Macrophage. Error bars indicate S.E.M. Statistical analysis by Mann Whitney *U* test. * = p ≤ .05; ** = p ≤ .01; *** = p ≤ .001; **** = p ≤ .0001.

Treatment with bezlotoxumab at 24-hours post CDI induced a similar level of protection from severe and systemic CDI complications to that seen with pre-infection prophylactic administration. *C. difficile* mediated weight loss was significantly reduced by 4.5% following bezlotoxumab treatment (~83.5% of starting weight in infected untreated mice compared to ~88% in treated infected mice; *p*= .0082; Figure 4a) and correlated with a 39% increase in survival (*p*= .0225; Figure 4b), when compared to infected mice treated with PBS. Bezlotoxumab treatment also alleviated systemic CDI symptoms, preventing the development of a scruffy coat and squinted eyes (*p*< .0001), and to a lesser degree, poor activity and breathing, when compared to PBS-treated infected mice (Figure 4c). Bezlotoxumab treatment of infected mice was able to prevent infection-mediated hypoglycemia and an increase in serum IL-6 when compared to infected PBS-treated mice (*p = *.0159; Supplementary Figure 2i, k), with treated, infected mice showing similar glucose and IL-6 levels to uninfected mice. By contrast, treatment of infected mice with bezlotoxumab did not significantly reduce serum urea when compared to infected PBS-treated mice, and had no effect on serum creatinine levels (Supplementary Figure 2 h, j). Of note, serum urea levels between infected mice given PBS prophylactically and as a treatment were different (Supplementary Figure 2 f and j), and may reflect that hydration of mice at 24-hours of infection, *via* the administration of 100 μl of PBS, might partly rescue mice from CDI-mediated uremia.

Similar to the prophylactic groups, treatment with bezlotoxumab 24-hours post CDI did not prevent severe colonic damage (*p*< .0001; Figure 4d, e), but prevented thymic atrophy and DP thymocyte depletion (*p*< .0001; Figure 4f, g). Furthermore, kidney inflammation was prevented in infected mice treated with bezlotoxumab, with glomerular macrophage counts similar to those seen in uninfected mice, compared to infected PBS-treated mice, in which high macrophage infiltration into the glomeruli (*p*= .0023; Figure 4h, i) was detected. This may account for the reduction in serum urea seen in infected bezlotoxumab-treated mice compared to infected PBS-treated mice (Supplementary Figure 2 j).

**Figure 5.**
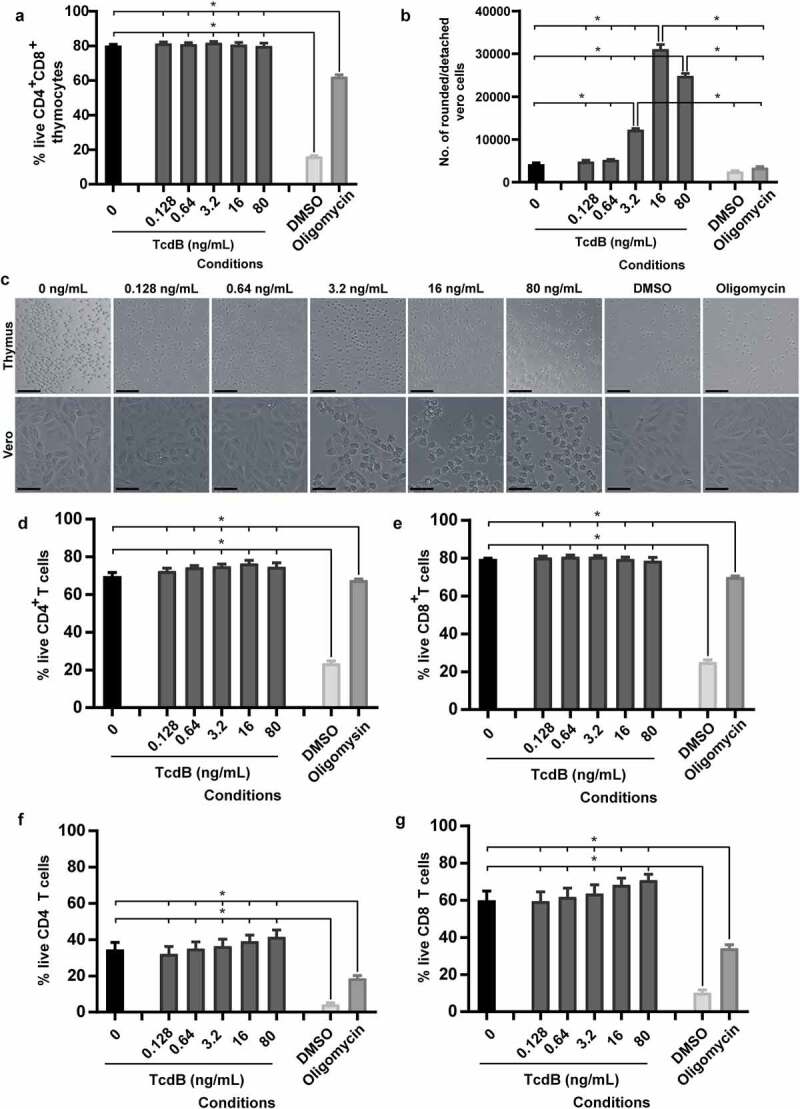
TcdB does not directly intoxicate thymocytes. Thymocytes and splenocytes (1x10^6^) isolated from three-month-old male C57BL/6 J mice and Vero cells (1x10^4^) were incubated with dilutions of recombinant *C. difficile* TcdB ranging from 80 ng/ml to 0.128 ng/ml, using media alone (negative), or 10% DMSO (positive) or 10% oligomycin (positive) as controls for thymocyte death. a) Thymocyte viability after TcdB incubation. b) Vero cell detachment as a measure of cell rounding after TcdB intoxication. c) Representative images of thymocytes and Vero cells following incubation with TcdB. Scale bar = 50 µm. Percentage of live thymic (d) CD4 T cells and (e) CD8 T cells and splenic (f) CD4 T cells and (g) CD8 T cells following incubation with TcdB; as measured by exclusion of both LIVE/DEAD™ Fixable Aqua (Invitrogen) and Propidium Iodide (BD Biosciences) stains. n = 4. Error bars indicate S.E.M. Statistical analysis by Mann Whitney *U* test. * = p ≤ .05.

The observation that bezlotoxumab prevented CDI-mediated thymic damage and thymocyte depletion may suggest that TcdB is acting directly on thymic cells. To investigate if TcdB directly interacts with and kills these cells, TcdB was applied directly to thymocytes and intoxication outcomes measured. Strikingly, direct exposure of isolated DP thymocytes to TcdB was unable to induce cell death, even at 80 ng/ml, despite high levels of toxin activity and cell rounding in control Vero cells (TcdB sensitive cell line; Figure 5a-c), with cell viability significantly above that detected for DMSO and oligomycin treated cells; both of which induce high and moderate leukocyte death respectively. This was supported by imaging of both DP thymocytes and Vero cells, where clear cell rounding was visible in Vero cells up to 3.2 ng/ml TcdB, confirming TcdB toxicity (Figure 5b). Similarly, TcdB was unable to directly kill naïve SP CD4^+^ and CD8^+^ T-cells, isolated from the thymus (Figure 5d, e) or spleen (Figure 5f, g). Thus, given the changes in serum cytokine levels detected following bezlotoxumab treatment but the absence of TcdB effects on thymocytes, we speculate that thymic atrophy and thymocyte depletion during CDI may occur in a similar manner to that seen with rabies virus, *Francisella tularensis* and *Salmonella enterica* serovar Typhimurium infections, where systemic increases in cytokines and cytokine-induced glucocorticoid hormone production facilitate thymic atrophy and thymocyte death.^38,49,50^ Blocking these toxin-mediated systemic complications during CDI may help to prevent the downstream impacts on vital organs, including the kidneys, which may improve overall disease outcomes.

## Discussion

Our work highlights the complexity of CDI and the ability of *C. difficile* to mediate disease beyond the gut, which is an overlooked aspect of this important enteric infection. Common presentations in severe CDI include ascites, bacteremia and toxemia, and can lead to organ failure in vital organs such as the brain, kidneys, liver, lungs or heart.^14–16^ However, the thymus is often overlooked in the examination of organ failure, even though many different pathogens can induce thymic disorganization or infect the thymus, leading to damage of this organ.^39,40,49,50^ Though not critical to everyday homeostatic processes, the thymus is specifically dedicated to the renewal and maintenance of the T cell population,^51^ which plays a key role in everyday survival and in responding to life-threatening infections. While *C. difficile* cells, spores and toxins may escape the gastrointestinal tract when toxin-mediated damage occurs,^17,27,52,53^ it is rare for extra-intestinal CDIs to occur. Thus, while *C. difficile* spores may be detectable in extra-intestinal organs, including the thymus,^53^ following infection mediated break down of cell-to-cell contacts within the colonic epithelium,^30,53^ *C. difficile* is unlikely to colonize these sites, primarily as they are oxygenated and do not support the growth of this strict anaerobe. However, the excessive and damaging inflammation of the intestine, increased serum cytokines and extra-intestinal pathologies seen in the WT infected mice suggest that a systemic escape of microbiota and toxins from the intestine occurs, resulting in these damaging outcomes.

The translocation of microbiota across a compromised intestinal barrier can lead to extra-intestinal pathologies, as seen in inflammatory bowel disease,^54,55^ or exacerbation of disease, as seen in chronic HIV infection.^56^ Increased systemic levels of inflammatory mediators may also play a role in thymic damage during CDI, in a similar way to that seen in other infections that lead to thymic atrophy.^49,50^ Specifically, *F. tularensis*,^49^ *T. cruzi*,^39^ *S. enterica* serovar Typhimurium^50^ and *M. avium*^57^ infections have been shown to induce thymic damage as a result of elevated glucocorticoid hormones and IL-6, as well as cytokines, including IFN-γ and TNF-α. Lastly, the escape of TcdA and TcdB from the intestine of infected pigs has been shown to induce damage in the lungs and ascites,^17,48,52^ and may account for some of the thymic and kidney damage seen with CDI, given that neutralization of TcdB by bezlotoxumab, both prophylactically and as a treatment, rescued infected mice from systemic disease. These findings are supported by other studies in which pre-clinical testing of intraperitoneal actoxumab-bezlotoxumab (TcdA/TcdB monoclonal antibody combination therapy) was also found to neutralize TcdB in the serum of CDI mice.^58^

Preventing systemic disease complications during CDI may be of particular interest when considering the high rates of recurrent infection that occur. Recurrent infection is likely to be influenced by a number of factors but the depletion of CD4^+^CD8^+^ thymocytes during thymic atrophy, detected in this study in infected mice, may also play a role in recovery from infection or susceptibility to recurrent disease. Infection recovery is characterized by the clearance of infection and the development of immunological memory facilitated by memory B and T cells, which should prevent the onset of recurrent disease, through rapid cellular proliferation and antibody production upon secondary exposure to the pathogen.^59,60^ Infection-induced thymic damage and DP thymocyte depletion results in fewer SP CD4 and CD8 naïve T cells migrating into the peripheral circulation of HIV infected mice^61^ and immature thymocytes escaping the thymus in *T. cruzi*^62^ and *Plasmodium berghei* infection.^63^ These phenomena may also be occurring during CDI.

Although it is not definitively known if thymic damage contributes to CDI relapse by reducing the pool of circulating naïve T cells that could interact with *C. difficile*, this hypothesis is supported by the observation that patients that experience CDI relapse often have lower levels of circulating immunoglobulins specific to *C. difficile* antigens, including the surface layer proteins (SLP), TcdA and TcdB.^64,65^ As the production of these immunoglobulins relies on T cell activation to induce proliferation of antibody-producing plasma cells and fine-tuning of antibody specificity and affinity,^66^ it is possible that a reduction in effective naïve T cells may result in a poor adaptive immune response toward *C. difficile*. This depletion of naïve T cells may contribute to ~25% of patients having at least one infection relapse, and a subset of these patients having additional relapse episodes, at ~45% and ~65% for second and third recurrences, respectively.^67^

Of further concern is the advanced age of most CDI patients (>65 years^25^), with immunosenescence contributing further to the depletion of naïve T cells and perhaps the capacity to mount an effective immune response during CDI. The results presented here thus raise the interesting proposition that CDI may compound immune aging and reduce any residual thymic function in older patients. This is in part supported by a recent study that shows CDI patients of advanced age (>65 years), have higher levels of mortality associated with CDI, and in particular systemic complications including sepsis and septic shock, and acute kidney failure.^68^ Although there is clear evidence that bezlotoxumab effectively reduces the rate of recurrence in CDI, the work presented here sheds some light on some of the novel mechanisms of action during acute disease that may contribute to reducing recurrence. In this regard, a recent study has shown that CDI patients with acute kidney damage have poor disease prognosis, linked to worsening of renal function and an increased risk of mortality.^69^ By preventing kidney inflammation during CDI, as seen when bezlotoxumab was administered as both a prophylactic and a treatment, it may be possible to reduce patient co-morbidities, and to reduce the risk of severe disease during CDI, by directly targeting the extraintestinal aspects of CDI.

Although progression of CDI into sepsis and septic shock is a rare complication of CDI, this disease profile is a major contributing factor to patient fatality. Detecting sepsis-like serum markers and disease symptoms in mice, and identifying therapeutics that prevent these outcomes, is an important outcome of the work described here as it provides new insights into this poorly characterized aspect of CDI. Administration of bezlotoxumab is already FDA approved, and is used primarily to prevent CDI recurrence. The work presented here shows that bezlotoxumab can prevent systemic disease pathologies and symptoms during acute infection, in particular thymic and kidney injury, and changes in serum glucose, urea and IL-6, and is supported by results published by others.^48,58^ Bezlotoxumab may therefore be a suitable adjunct therapy for cases of severe primary CDI, and may prevent the onset of systemic disease complications that may worsen patient outcomes. Adjunct therapies such as pooled human intravenous immunoglobulin (IVIG) have shown efficacy in reducing *C. difficile* disease and aiding in disease resolution (reviewed in^70^), however they vary in formulation and in TcdA/B antibody levels; thus, bezlotoxumab may be a more suitable treatment alternative. By detecting these changes early in disease, timely intervention can occur, which may help prevent the onset of severe symptoms and improve the current mortality rate of CDI, at roughly 20,000 deaths each year in the U.S.A alone.^71^

## Supplementary Material

Supplemental Material

## Data Availability

The data that support the findings of this study are available from the corresponding author, DL, upon reasonable request.
