# Supplementary material for: Bezlotoxumab prevents extraintestinal organ damage induced by Clostridioides difficile infection
Source: Gut Microbes. 2022 Aug 31;14(1):2117504. doi: 10.1080/19490976.2022.2117504 (PMC9450906; doi:10.1080/19490976.2022.2117504)
Supplement: Supplemental Material [file KGMI_A_2117504_SM1322.zip › Mileto et al CDI Systemic and Treatment_Supplementary Figures.docx]

# Supplementary Figures


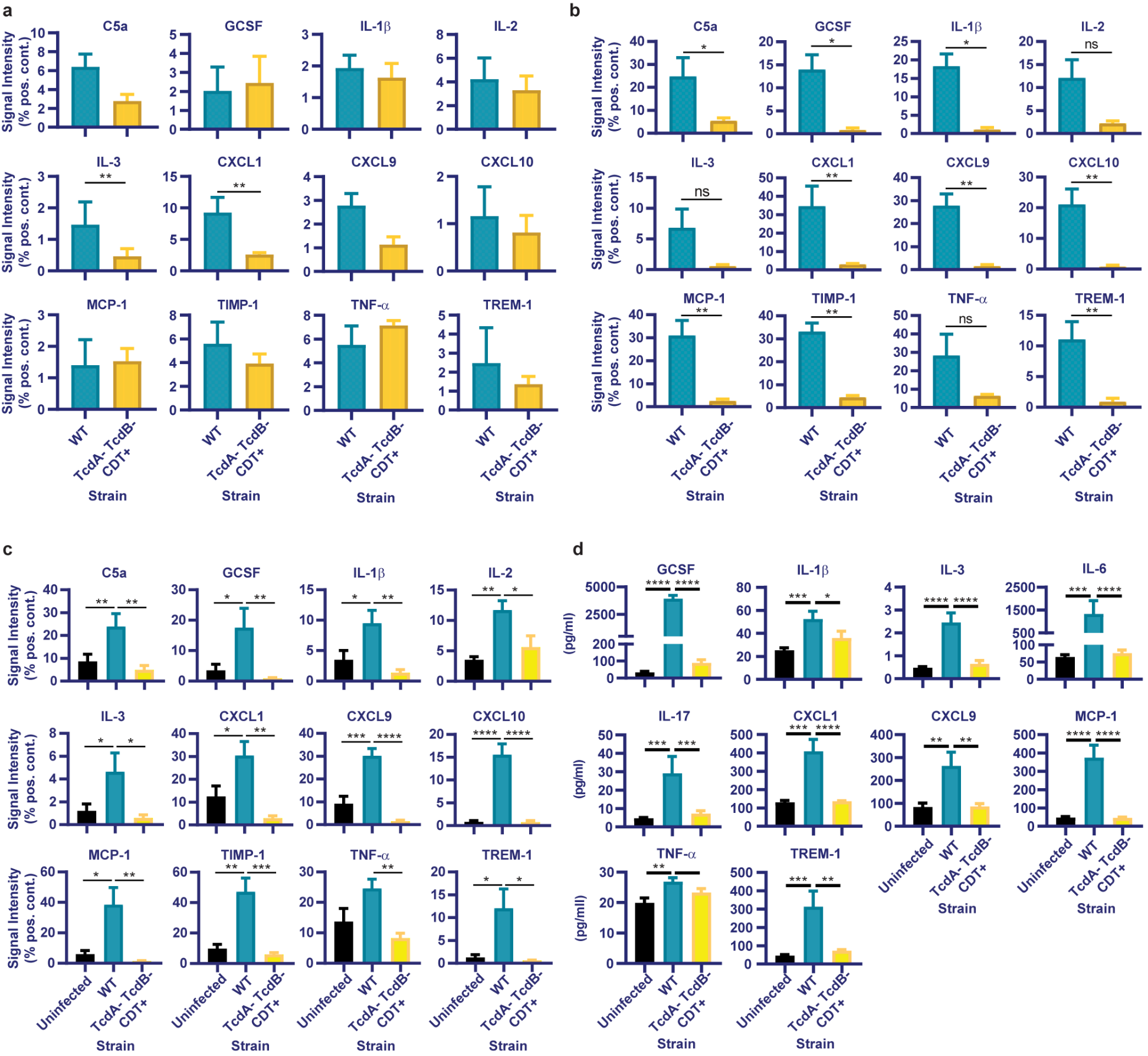


Supplementary Figure 1. Toxigenic M7404 (WT) *C. difficile* infection induces changes in colonic and serum cytokine and chemokine expression with progression of infection. Groups of C57BL/6J mice were infected with either M7404 (WT; n=10) or TcdA^-^TcdB^-^CDT^+^ *C. difficile* (n=10) or uninfected (n=5). Colonic tissues lysates were collected at 12- (A), 24- (B) or 48-hours (C) post-infection and analyzed for cytokine and chemokine expression as signal intensity as a percentage of the positive control. D) Serum was collected at 48-hours post infection and analyzed for cytokine and chemokine levels. Error bars indicate S.E.M. * = p ≤ 0.05; ** = p ≤ 0.01; *** = p ≤ 0.001; **** = p ≤ 0.0001.


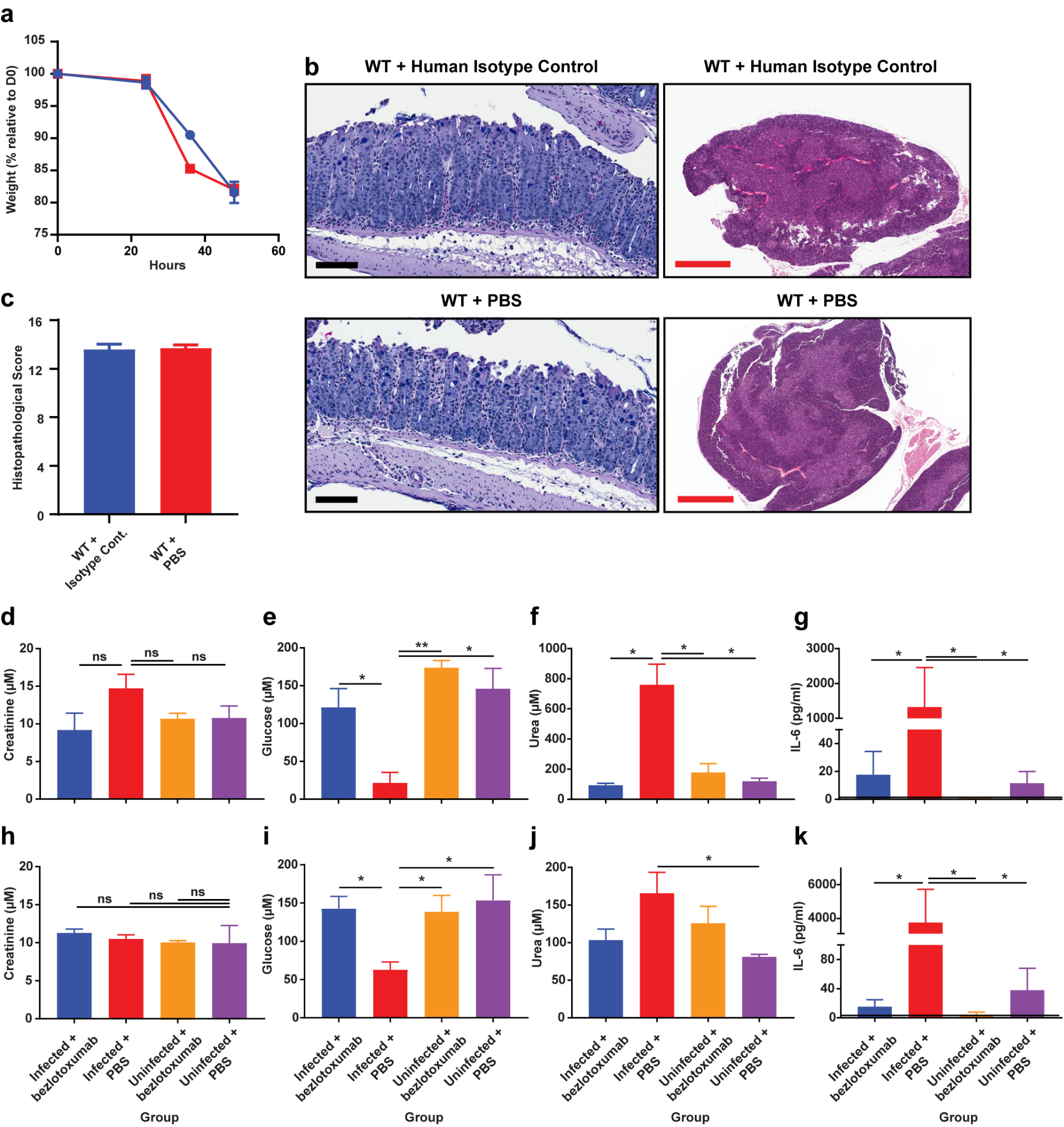


Supplementary Figure 2. Toxigenic M7404 (WT) *C. difficile* infection induces hypoglycemia, uremia, and elevated IL-6 at the peak infection, which is prevented by bezlotoxumab intervention. C57BL/6J mice (n=5) were infected with either M7404 (WT) *C. difficile* or left uninfected. Mice were administered either a human isotype control (IgG1) or PBS 24-hours prior to infection to assess the contribution of non-specific antibodies in preventing *C. difficile* mediated extra-intestinal disease. No difference was seen between groups when assessed for (A) weight-loss, (B) colonic and thymic pathology and (C) colonic histopathological scores. Bezlotoxumab or PBS was administered either 24-hours prior to infection (Prophylaxis; D-G) or 24-hours post infection (Treatment; H-K). Serum was collected at 48-hours post infection and analyzed for (D/H) Creatinine, (E/I) Glucose, (F/J) Urea and (G/K) IL-6. Error bars indicate S.E.M. * = p ≤ 0.05; ** = p ≤ 0.01.
