# Supplementary figures and images for: Bezlotoxumab prevents extraintestinal organ damage induced by Clostridioides difficile infection
Source: Gut Microbes. 2022 Aug 31;14(1):2117504. doi: 10.1080/19490976.2022.2117504 (PMC9450906; doi:10.1080/19490976.2022.2117504)

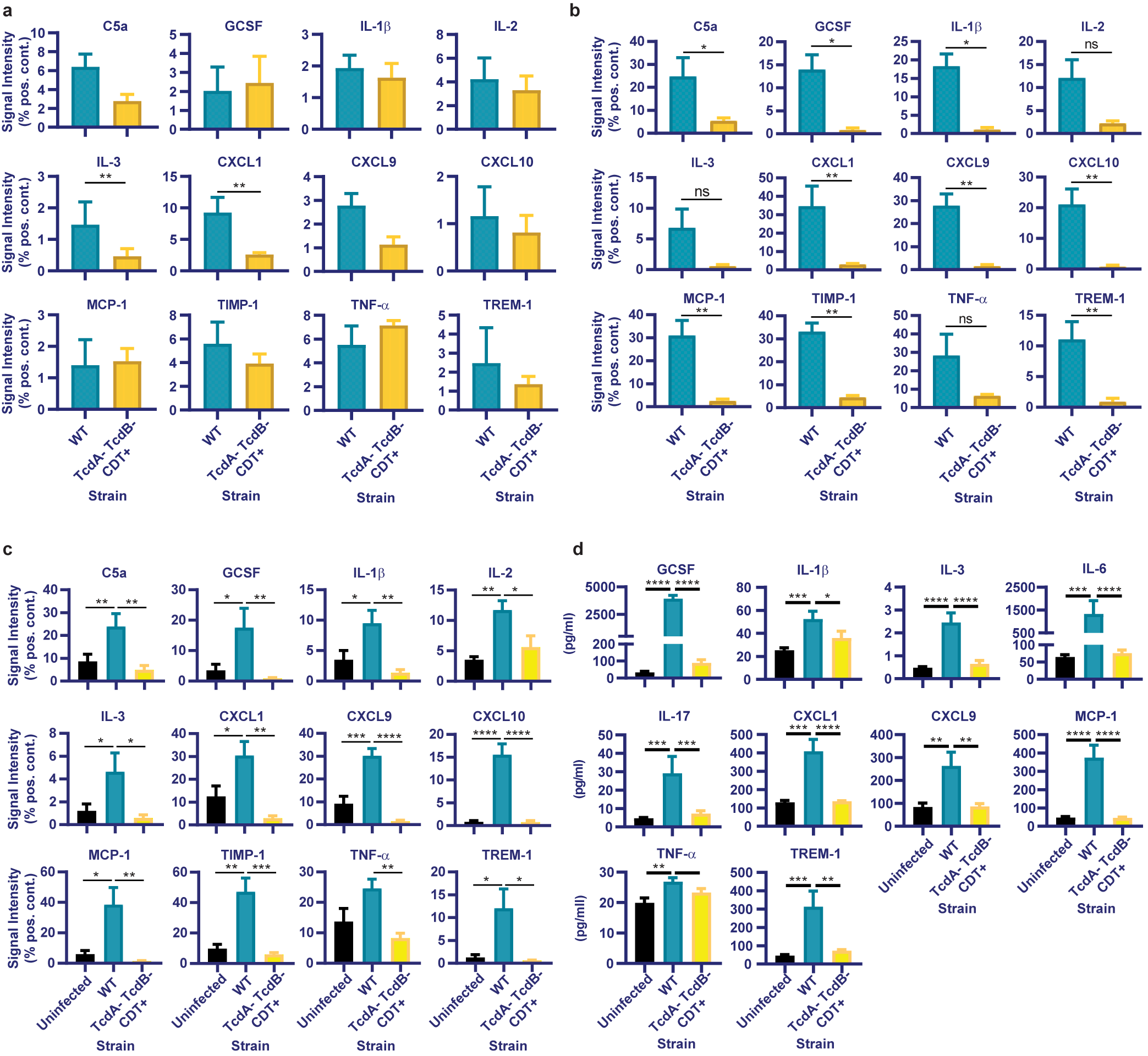

Supplement: Supplemental Material [file KGMI_A_2117504_SM1322.zip › Fig S1.tif]

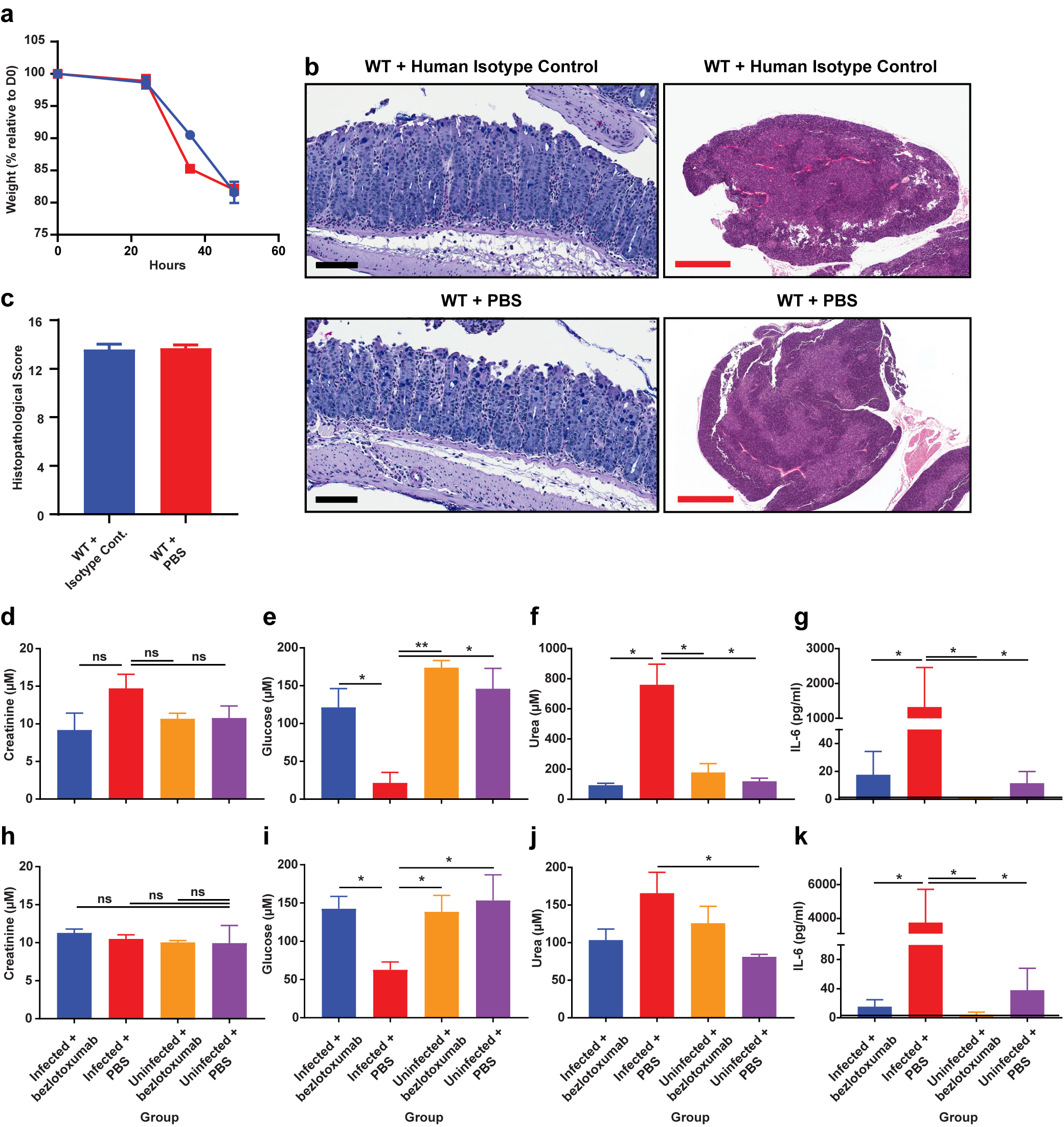

Supplement: Supplemental Material [file KGMI_A_2117504_SM1322.zip › Fig S2.tif]
